# Unreduced Megagametophyte Production in Lemon Occurs via Three Meiotic Mechanisms, Predominantly Second-Division Restitution

**DOI:** 10.3389/fpls.2017.01211

**Published:** 2017-07-12

**Authors:** Houssem Rouiss, José Cuenca, Luis Navarro, Patrick Ollitrault, Pablo Aleza

**Affiliations:** ^1^Centro de Citricultura y Producción Vegetal, Instituto Valenciano de Investigaciones Agrarias Moncada, Valencia, Spain; ^2^Unité Mixte de Recherche Amélioration Génétique et Adaptation des Plantes (UMR Agap), Centre de Coopération Internationale en Recherche Agronomique pour le Développement (CIRAD), Station de Roujol Petit-Bourg, Guadeloupe, France

**Keywords:** *Citrus*, unreduced gametes, meiotic restitution, second-division restitution (SDR), first-division restitution (FDR), post-meiotic genome doubling (PMD) mechanisms, seedlessness

## Abstract

Unreduced (2n) gametes have played a pivotal role in polyploid plant evolution and are useful for sexual polyploid breeding in various species, particularly for developing new seedless citrus varieties. The underlying mechanisms of 2n gamete formation were recently revealed for *Citrus reticulata* but remain poorly understood for other citrus species, including lemon (*C. limon* [L.] Burm. f.). Here, we investigated the frequency and causal meiotic mechanisms of 2n megagametophyte production in lemon. We genotyped 48progeny plants of two lemon genotypes, “Eureka Frost” and “Fino”, using 16 Simple Sequence Repeat (SSR) and 18 Single Nucleotide Polymorphism (SNP) markers to determine the genetic origin of the progenies and the underlying mechanisms for 2n gamete formation. We utilized a maximum-likelihood method based on parental heterozygosity restitution (PHR) of centromeric markers and analysis of PHR patterns along the chromosome. The frequency of 2n gamete production was 4.9% for “Eureka Frost” and 8.3% for “Fino”, with three meiotic mechanisms leading to 2n gamete formation. We performed the maximum-likelihood method at the individual level via centromeric marker analysis, finding that 88% of the hybrids arose from second-division restitution (SDR), 7% from first-division restitution (FDR) or pre-meiotic doubling (PRD), and 5% from post-meiotic genome doubling (PMD). The pattern of PHR along LG1 confirmed that SDR is the main mechanism for 2n gamete production. Recombination analysis between markers in this LG revealed partial chiasma interference on both arms. We discuss the implications of these restitution mechanisms for citrus breeding and lemon genetics.

## Introduction

The exact area of origin of lemon (*Citrus limon* [L.] Burm. f.) is uncertain, but this crop likely originated in Northern India and South East China or in northern Myanmar (Curk et al., 2016). Molecular analyses indicate that this species resulted from direct hybridization between *C. aurantium* L. (sour orange) as the female parent and *C. medica* L. (citron) as the male parent (Nicolosi et al., 2000; Froelicher et al., 2011; García-Lor et al., 2013; Curk et al., 2016).

The Mediterranean Basin is a major area of lemon production, accounting for 48% of production worldwide (Duportal et al., 2013). Turkey is the most important lemon-producing country in this area (annual production >1,000,000 tons), followed by Spain (900,000 tons) and Italy (500,000 tons) (Martín and González, 2014). Seedless lemons with high organoleptical qualities and resistance to important diseases, such as Mal secco caused by *Phoma tracheiphila*, are in high demand by consumers and growers (Uzun et al., 2008; Migheli et al., 2009; Pérez-Tornero et al., 2012). Several lemon-breeding programs worldwide are focused on meeting this demand (Calabrese et al., 2000; Recupero et al., 2005; Spiegel-Roy et al., 2007; Uzun et al., 2008; Pérez-Tornero et al., 2012), despite the difficulties imposed by the high heterozygosity and low genetic variation of this species (Krueger and Navarro, 2007).

In *Citrus*, diploidy is the general rule, with a basic chromosome number x = 9 (Krug, 1943), although triploid and tetraploid genotypes are present in the citrus germplasm (Lee, 1988). Triploid citrus plants are currently being produced in various breeding programs for the development of new seedless commercial citrus varieties (Starrantino and Recupero, 1981; Ollitrault et al., 2008; Grosser et al., 2010; Navarro et al., 2015). Triploid citrus plants can be recovered from interploid hybridizations, 2x X 4x and 4x X 2x (Esen and Soost, 1973b; Cameron and Burnett, 1978; Starrantino and Recupero, 1981; Ollitrault et al., 2008; Grosser and Gmitter, 2011; Aleza et al., 2012a,b; Navarro et al., 2015), or by 2x X 2x sexual hybridizations as a consequence of unreduced (2n) gamete formation (Esen and Soost, 1971, 1973a; Ollitrault et al., 2008; Aleza et al., 2010a; Cuenca et al., 2015; Navarro et al., 2015). The sexual 2x X 2x hybridization strategy was used by Geraci et al. (1975) and Esen and Soost (1975) to obtain triploid progenies using “Lisbon” and “Eureka” lemons as female parents. Viloria and Grosser (2005) and Recupero et al. (2005) recovered progenies of triploid lemon-like hybrids via 2x X 4x sexual hybridizations. Pérez-Tornero et al. (2012) started a lemon-breeding program in 2008 aimed at obtaining triploid hybrids of higher quality than “Fino” and “Verna” lemons, the most important lemon varieties in Spain.

The frequency of 2n female gametes, an intrinsic characteristic of citrus genotypes, can vary from <1% to over 20% (Esen and Soost, 1971; Ollitrault et al., 2008). For *C. limon*, 1 and 5% of triploid progenies were recovered from 2x X 2x sexual hybridizations using “Lisbon” and “Eureka” lemons as the female parents, respectively (Esen and Soost, 1975; Geraci et al., 1975). Moreover, Pérez-Tornero et al. (2012) obtained 5.8 to 8.6% of triploid hybrids from a 2x X 2x cross between “Verna” and “Fino” genotypes. Various meiotic aberrations can result in unreduced gamete formation. First-division restitution (FDR) and second-division restitution (SDR) are the predominant mechanisms of 2n gamete formation in plants (De Storme and Geelen, 2013). These gametes are produced as a consequence of the failure of the first or second meiotic division, respectively, leading to the formation of restitution nuclei with a somatic chromosome number (Mendiburu and Peloquin, 1976; Park et al., 2007). As a result, FDR and SDR have different genetic implications. FDR 2n gametes contain non-sister chromatids, which in the absence of crossover maintain the parental heterozygosity. When crossing over occurs, the parental heterozygosity restitution (PHR) rates vary from 100% for loci close to the centromere to 60–70% for loci far from the centromere, depending on the level of chromosome interference (Cuenca et al., 2011). For SDR, the 2n gametes contain two sister chromatids, which reduces the parental heterozygosity level (Bastiaanssen et al., 1998; Cuenca et al., 2011; De Storme and Geelen, 2013). When crossing over occurs, the PHR rate varies from 0% for loci close to the centromere to 60–75% for loci far from the centromere, depending on the level of chromosome interference (Cuenca et al., 2011). SDR is the dominant mechanism involved in the origin of unreduced female gametes in clementines and mandarins (Luro et al., 2004; Cuenca et al., 2011, 2015; Aleza et al., 2016). Ferrante et al. (2010) reported that FDR is the main mechanism for unreduced female gamete formation in lemon. However, their results were based on the analysis of only a few individuals with few markers and without previous knowledge of centromere location.

Other mechanisms leading to unreduced gamete formation have been described, such as pre-meiotic (PRD) and post-meiotic genome doubling (PMD). Although, PMD was identified in potato (Bastiaanssen et al., 1998), both mechanisms have only rarely been documented in plants (De Storme and Geelen, 2013). PRD produces 2n gametes equivalent to the meiosis of doubled diploid genotypes. Therefore, PHR depends mainly on the chromosomal preferential pairing rate (Stift et al., 2008), which should vary between 66% for fully tetrasomic meiosis to 100% for fully disomic meiosis. Little variation can occur along the chromosome due to double reduction events. In the case of PMD, haploid gametes undergo an extra round of genome duplication, leading to the formation of fully homozygous 2n gametes (Bastiaanssen et al., 1998; Ramanna and Jacobsen, 2003; De Storme and Geelen, 2013; Cuenca et al., 2015). Thus, 100% homozygosity for all loci is expected among the 2n gametes (Ramanna and Jacobsen, 2003). SDR can also produce 100% homozygosity for centromeric markers, but not for telomeric ones (Cuenca et al., 2011). Therefore, in order to distinguish between both mechanisms, Cuenca et al. (2015) genotyped telomeric loci to determine whether diploid gametes fully homozygous for centromeric markers resulted from PMD or SDR. Moreover, Bastiaanssen et al. (1998) identified 2n female gametes of potatoes fully homozygous for RFLP markers. The evidence for recombination between alleles originating from the two ancestors of the parent producing 2n gametes indicated that these gametes originated from PMD.

Molecular marker analyses can be used to estimate the PHR rates for diploid gametes in polyploid progenies and, therefore, to identify the mechanisms underlying unreduced gamete formation (Cuenca et al., 2011). Cuenca et al. (2015) took advantage of known citrus centromere locations (Aleza et al., 2015) to develop a maximum-likelihood method that distinguishes between SDR and FDR mechanisms at both the population and individual levels based on the PHR patterns of unlinked markers located close to the centromeres of different chromosomes.

In the current study, we analyzed the frequencies of 2n gamete formation and the causal meiotic mechanisms leading to 2n gamete formation in two varieties of lemon, “Eureka Frost” and “Fino”, through genetic analyses of triploid and tetraploid hybrids recovered from 2x X 2x and 2x X 4x sexual hybridizations. We used the maximum-likelihood method based on centromeric molecular markers in conjunction with a telomeric loci study and analysis of the pattern of PHR variation along linkage group 1 (LG1) to identify the mechanisms underlying unreduced gamete formation at the individual and population level. Crossover interference was also analyzed. We discuss the implications for breeding programs based on sexual polyploidization.

## Materials and methods

### Plant material

Triploid and tetraploid citrus hybrids were obtained via 2x X 2x and 2x X 4x sexual hybridizations using diploid “Eureka Frost” and “Fino” lemon genotypes as female parents pollinated with diploid “Fortune” mandarin (*C. clementina* x *C. tangerina*) and *C. ichangensis* Swing and tetraploid *C. macrophylla* Wester. Flowers in pre-anthesis were emasculated, pollinated, and enclosed with a cloth bag. A total of 115 “Eureka Frost” lemon flowers were pollinated, including 55 with “Fortune” mandarin (named EuFor) and 60 with *C. ichangensis* (named EuIch), while 15 “Fino” lemon flowers were pollinated with tetraploid *C. macrophylla* (named FinMac). The detailed methods used for plant recovery via *in vitro* embryo rescue and ploidy level analysis via flow cytometry can be found in Aleza et al. (2010a; 2010b; 2012a; 2012b).

### Genotyping of progenies using Simple Sequence Repeat (SSR) and Single Nucleotide Polymorphism (SNP) markers

The male and female parents and 48 hybrids were genotyped using 34 molecular markers (16 Simple Sequence Repeats [SSRs] and 18 Single Nucleotide Polymorphisms [SNPs]) showing heterozygosity for the lemon genotypes and polymorphism with the male parents. These markers are distributed across all LGs of the clementine genetic map (Ollitrault et al., 2012a). Detailed information about the markers is provided in Table 1.

**Table 1:** Information about the molecular markers used in this study, including GenBank accession numbers, genetic distances, noted alleles, and references.

***Locus***	**Phytozome/Gene Bank Accession**	**LG**	**GMP (cM)**	**DC**	**Noted alleles**^a^					**Used to identify th**				**Bibliographic references**
					**“Eureka Frost”**	**“Fino”**	**C. *macrophylla***	**C. *ichangensis***	**Fortune**	**2n gamete origin**	**LOD**	**HR pattern LG1**	**PMD**	
CIBE6126	ET084980	1	2.69	57.97	218-220	218-220	218-218	223-230	230-244			1		Ollitrault et al., 2010
CiC2110-02	ET099643	1	29.61	31.05	A-C	A-C	A-A	A-A	C-C			1		Ollitrault et al., 2012b
mCrCIR06B05	AM489744	1	50.27	10.39	187-199	187-199	187-187	185-185	187-187			1		Froelicher et al., 2008
MEST001	DY262452	1	70.61	9.95	176-192	176-192	187-199	190-190	172-172	1	1	1		Luro et al., 2008
CiC5950-02	ET083949	1	91.37	30.71	A-G	A-G	A-A	A-A	G-G			1		Ollitrault et al., 2012b
MEST431	DY291553	1	119.00	58.34	331-348	331-348	345-348	340-342	331-331			1		García-Lor et al., 2012
JK-CAC15	none	2	43.51	13.36	160-163	160-163	152-160	160-160	151-163		1	1		Kijas et al., 1997
mCrCIR03C08	FR677576	2	82.19	25.32	210-214	210-214	198-214	210-214	226-226				1	Cuenca et al., 2011
CiC3712-01	ET079481	2	93.92	37.05	AC	AC	AA	AA	AA				1	Ollitrault et al., 2012b
JK-TAA41	none	2	131.86	74.99	145-150	145-150	132-154	147-162	137-147	1			1	Kijas et al., 1997
3P165889	Ciclev10023360 m.g	3	1.00	89.59	AG	AG	AA	AA	AG				1	Curk et al., 2015
3P11355960	Ciclev10023509 m.g	3	88.50	2.09	AG	AG	AG	AA	AA		1			Curk et al., 2015
CiC1459-02	ET073328	3	118.06	27.47	AC	AC	CC	CC	AA				1	Ollitrault et al., 2012b
MEST131	DY276912	3	179.33	88.74	135-147	135-147	147-147	135-141	141-141	1				García-Lor et al., 2012
CiC4240-04	ET106812	4	7.09	9.05	AG	AG	GG	GG	AG		1			Ollitrault et al., 2012b
mCrCIR07D06	FR677581	4	16.33	0.19	164-168	164-168	168-168	166-178	166-168		1			Cuenca et al., 2011
mCrCIR03G05	FR677578	4	75.06	58.92	226-229	226-229	218-218	218-218	199-228				1	Cuenca et al., 2011
5p22687304	Ciclev10001185 m.g	5	21.00	2.12	AC	AC	AC	AA	AA		1			Curk et al., 2015
CiC5842-02	ET083106	5	77.34	54.22	AC	AC	CC	CC	AC				1	Ollitrault et al., 2012b
CiC4356-06	ET107540	6	6.21	0.19	CT	CT	CT	CC	CT		1			Ollitrault et al., 2012b
6p7496245	Ciclev10013603 m.g	6	6.50	0.10	GC	GC	GC	CC	CC		1			Curk et al., 2015
LapXcF238	EU719653	6	11.00	4.60	GC	GC	GG	GG	GC		1			Ollitrault et al., 2012b
MEST488	DY297637	6	68.48	62.08	119-133	119-133	119-127	143-153	147-155				1	García-Lor et al., 2012
JK-TAA1	none	6	93.49	87.09	170-180	170-180	146-162	146-162	160-164	1				Kijas et al., 1997
mCrCIR03B07	FR677573	7	83.39	13.04	269-273	269-273	273-277	267-282	267-282		1			Cuenca et al., 2011
CiC3674-02	ET079224	7	23.56	72.87	AG	AG	AA	AA	AG				1	Ollitrault et al., 2012b
8P18684429	Ciclev10028449 m.g	8	56.00	1.79	CT	CT	CT	CC	CC		1			Curk et al., 2015
8P16570424	Ciclev10029557 m.g	8	50.00	4.21	AG	AG	GG	GG	AA		1			Curk et al., 2015
8P2427684	Ciclev10029965 m.g	8	20.69	33.52	AT	AT	TT	TT	AA				1	Curk et al., 2015
Ci02B07	AJ567403	9	0.00	52.16	164-170	164-170	170-172	162-172	178-182	1				Froelicher et al., 2008
CiC4876-07	ET080580	9	2.69	49.47	AT	AT	TT	TT	AT				1	Ollitrault et al., 2012b
9p4699283	Ciclev10005777 m.g	9	50.00	2.16	AG	AG	AG	AA	AA		1			Curk et al., 2015
CIBE3966	ET105040	9	52.27	0.11	106-118	106-118	118-N	106-118	106-118		1			Ollitrault et al., 2010
Ci07C09	AJ567410	9	53.00	0.84	242-250	242-250	242-252	240-242	242-242		1			Froelicher et al., 2008

a *Noted alleles. The numbers indicate the size of alleles in nucleotides for SSR markers and letters correspond to SNP markers alleles. N. Indicate null alleles. LG, linkage group; GMP, genetic map position; DC, distance to the centromere*.

Genomic DNA was isolated using a Plant DNeasy kit from Qiagen Inc. (Valencia, CA, USA) following the manufacturer's protocol. PCR amplifications using 16 SSR markers were performed using a Thermocycler rep gradient S (Eppendorf®) in a 10 μL final volume containing 0.8 U of Taq DNA polymerase (Fermentas®), 2 ng/μL citrus DNA, 0.2 mM welled (Sigma®) dye-labeled forward primer, 0.2 mM non-dye-labeled reverse primer, 0.2 mM of each dNTP, 10 × PCR buffer, and 1.5 mM MgCl_2_. The PCR protocol was as follows: denaturation at 94°C for 5 min followed by 40 cycles of 30 s at 94°C, 1 min at 50 or 55°C, and 45 s at 72°C; and a final elongation step of 4 min at 72°C. Capillary electrophoresis was carried out using a CEN™ 8,000 Genetic Analysis System (Beckman Coulter Inc.). The PCR products were initially denatured at 90°C for 2 min, injected at 2 kV for 30 s, and separated at 6 kV for 35 min. Alleles were sized based on a DNA size standard (400 bp). Genome Lab™ Gap v.10.0 genetic analysis software was used for data collection. Allele dosage was calculated using the MAC-PR (microsatellite DNA allele counting-peak ratio) method (Esselink et al., 2004), validated in citrus by Cuenca et al. (2011).

Triploid and tetraploid hybrids were also genotyped with 18 SNP markers using KASPar™ technology by LGC Genomics (www.lgcgenomics.com). The KASPar Genotyping System is a competitive, allele-specific dual Förster Resonance Energy Transfer (FRET)-based assay for SNP genotyping. Primers were directly designed by LGC Genomics Company based on the SNP locus-flanking sequence (~50 nt on each side of the SNP). SNP genotyping was performed using the KASPar technique. A detailed description of specific conditions and reagents can be found in Cuppen (2007). Identification of allele doses in heterozygous triploid and tetraploid hybrids was carried out based on the relative allele signals, as described by Cuenca et al. (2013a) and Aleza et al. (2015).

### Identification of the parent producing the unreduced gamete and inference of the unreduced gamete genotype

For triploid and tetraploid hybrids, the 2n gamete origin was determined by identifying the parent that passed double genetic information onto the hybrid. Markers with total differentiation between the parents (A_1_A_1_ x A_2_A_2_A_2_A_2_, A_1_A_2_ x A_3_A_3_A_3_A_3_, and A_1_A_2_ x A_3_A_3_A_4_A_4_ in 2x X 4x crosses) for tetraploids and (A_1_A_1_ x A_2_A_2_, A_1_A_2_ x A_3_A_3_, and A_1_A_2_ x A_3_A_4_ in 2x X 2x crosses) for triploids were the best allelic configurations, as described by Aleza et al. (2015) and Cuenca et al. (2015). Indeed, conclusive results can be obtained using only one marker, as was the case for FinMac hybridization using the JK-TAA41 SSR marker. However, for EuFor and EuIch hybridizations, more than one marker had to be analyzed to observe both alleles from the female parent at least once for each hybrid. The SSRs JK-TAA1, JK-TAA41, and MEST131 were used for EuFor hybridization, and JK-TAA1, JK-TAA41, MEST001, and Ci02B07 were used for EuIch.

Once the female origin of the diploid gamete was demonstrated, inference of the allelic configurations of the 2n gametes from hybrid genotyping was performed as described by Cuenca et al. (2011). In the case of FinMac tetraploid hybridization, for the A_1_A_2_ × A_3_A_3_A_3_A_3_ and A_1_A_2_ × A_3_A_3_A_4_A_4_ allelic configurations, the genotype of the unreduced gamete was deduced directly from observation of both A_1_ and A_2_ alleles in the tetraploid hybrids. However, when the male and female parents shared one allele (A_1_A_2_ × A_1_A_1_A_1_A_1_ and A_1_A_2_ × A_1_A_1_A_3_A_3_), for the tetraploid hybrids that inherited the common allele (A_1_), inference of the unreduced female gamete structure was carried out based on the estimated allele dosage in the tetraploid hybrid.

In the case of triploid hybrids obtained from EuFor and EuIch hybridizations, for A_1_A_2_ x A_3_A_3_ and A_1_A_2_ x A_3_A_4_, the genotype of the 2n gamete was deducted directly from the triploid hybrid structure. When the male and female genitors shared one allele (A_1_A_2_ x A_2_A_2_ and A_1_A_2_ x A_2_A_3_), the 2n female gamete structure for the triploid hybrids with a common allele from the male genitor was inferred from the estimated allele dosage in the triploid hybrid.

### Identification of the mechanism underlying unreduced gamete formation

For the EuFor and EuIch progenies, nine SSR and SNP molecular markers within 20 cM of the centromere (Aleza et al., 2015) located in all nine LGs of the clementine genetic map (Ollitrault et al., 2012a) were genotyped to determine the mechanism of 2n gamete formation for each population. The molecular markers used included MEST001, JK-CAC15, 3p11355960, mCrCIR07D06, 5p22687304, 6p7496245, mCrCIR03B07, 8p18684429, and Ci07C09 for EuFor hybridization and MEST001, JK-CAC15, 3p11355960, mCrCIR07D06, 5p22687304, CiC4356-06, mCrCIR03B07, 8p18684429, and 9p4699283 for EuIch hybridization. For FinMac hybridization, seven molecular markers distributed in seven LGs were used, including MEST001, JK-CAC15, CiC4240-04, LapXcF238, mCrCIR03B07, 8P16570424, and CIBE3966.

To distinguish between the SDR and FDR hypotheses, the maximum-likelihood method based on the LOD score test described by Cuenca et al. (2015) was employed. LODs >2 were considered to be significant for SDR, those < −2 were considered to be significant for FDR, and those between 2 and −2 were considered not to be significant. To compare the SDR hypothesis with the PRD hypothesis using LOD scores, we considered the minimum value of 66% of PHR as the theoretical value for the PRD hypothesis.

Additionally, a set of six SSR and SNP molecular markers distributed along LG1 were used to analyze PHR evolution, including SSR markers MEST001, mCrCIR06B05, CIBE6126, and MEST431 and SNP markers CiC5950-02 and CiC2110-02. Moreover, a complementary experiment was performed to differentiate between PMD and SDR mechanisms using 11 telomeric molecular markers in LG2 to LG9. These included SSR markers mCrCIR03C08, JK-TAA41, MEST488 and mCrCIR03G05 and SNP markers CiC4876-07, CiC3674-02, CiC5842-02, CiC1459-02, CiC3712-01, 3p165889, and 8p2427684. The marker positions and distances to the centromeres of each LG are shown in Table 1.

### Interference analysis

Taking into account the centromere position, three-point linkage mapping was performed to estimate chiasma interference for each chromosome arm of chromosome I. The centromere was used as the first point, and two markers were selected on each arm (MEST001 and MEST431 on one arm and mCrCIR06B05 and CIBE6126 on the other arm). The chromosome interference coefficient (IC) is defined as follows (as per Griffiths et al., 1996):

(1) $$\begin{array}{l} \text{IC}=1-\left[\frac{rd}{r_{{CM}_{1}}\cdot r_{M_{1}M_{2}}}\right] \end{array}$$

Where *r*_*CM*1_ indicates the observed recombination rate (heterozygous to homozygous and vice versa) between the centromere and locus 1; *r*_*M*1*M*2_, the observed recombination between locus1 and 2; and rd, the observed rate of double recombination between the centromere and locus 2.

## Results and discussion

### Parental origin of recovered plants and frequencies of unreduced gametes

For sexual hybridizations between “Eureka Frost” lemon as the female parent and “Fortune” mandarin and *C. ichangensis* as the male parents, the average fruit set was 45.5 and 36.7%, respectively (Table 2), yielding 250 and 464 seeds, respectively, from both hybridizations. We classified the seeds by size, since, according to Aleza et al. (2010a), seed size is highly correlated to ploidy level. While small seeds are expected to contain triploid embryos, tetraploids are generally observed in normal size seeds. Thus, we selected 45 and 40 small seeds from the EuFor and EuIch hybridizations, respectively, for plant regeneration by embryo rescue.

**Table 2:** Plant regeneration and ploidy level analysis of plants recovered from “Eureka Frost” X “Fortune” mandarin (EuFor), “Eureka Frost” X *C. ichangensis* (EuIch), and “Fino” X *C. macrophylla* (FinMac).

**Hybridization**	**Pollinated flowers**	**Fruits set**	**Total number of seeds**	**Normal seeds**	**Undeveloped seeds**	**Small seeds**	**Cultured embryos**	**Recovered plants**	**Diploid plants**	**Triploid plants**	**Tetraploid plants**
**EuFor**	55	25	464	419	0	45	54	53	32	21	0
**EuIch**	60	22	250	210	0	40	40	35	21	14	0
**FinMac**	15	8	156	36	154	36	36	36	0	23	13

From the 45 small seeds obtained in the EuFor hybridization, 54 embryos were cultured *in vitro*, with an average of 1.2 embryos per seed, indicating a low rate of polyembryony in “Eureka” lemon. Of the 53 plantlets recovered, 32 were diploid and 21 triploid. All 40 small seeds recovered from the EuIch hybridization contained only a single embryo. Of the 35 plants regenerated, 21 were diploid and 14 were triploid. For the FinMac 2x X 4x sexual hybridization, the average fruit set was 53.3%, and 36 normal seeds were obtained according to the size classification of Aleza et al. (2012b). Of the 36 plants recovered, 23 were triploid and 13 were tetraploid (Table 2).

**Figure 1:**
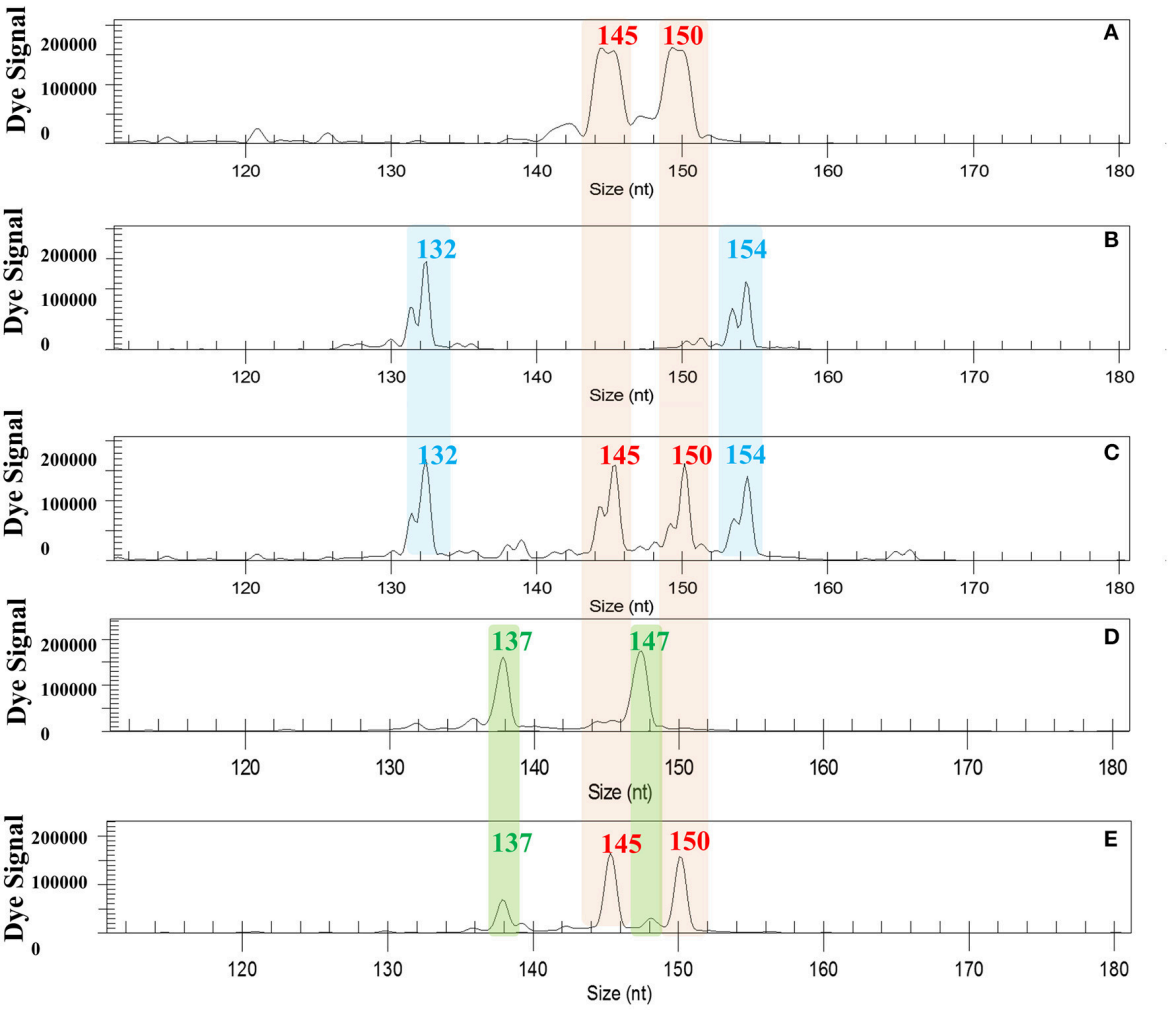
Electropherograms of a triploid and a tetraploid hybrid recovered from EuIch and FinMac hybridizations using SSR marker JK-TAA 41. **(A)** “Fino” and “Eureka Frost” lemons displayed the same allelic configuration fr this marker; **(B)** *C. macrophylla*; **(C)** tetraploid hybrid with four different alleles from “Fino” X 4x *C. macrophylla* hybridization. **(D)** *C. ichangensis*. **(E)**. Triploid hybrids with two alleles from the female parent “Eureka Frost” lemon and one from the male parent *C. ichangensis*. nt: nucleotides.

To determine which parent passed double genetic information onto the hybrids, we genotyped triploid hybrids recovered from the 2x X 2x hybridizations using markers that displayed total allelic differentiation between “Eureka Frost” lemon and the male parents, “Fortune” mandarin and *C. ichangensis* (Figure 1): SSR markers JK-TAA1, JK-TAA41, and MEST131 for the EuFor hybridization and SSR markers JK-TAA1, JK-TAA41, MEST001, and Ci02B07 for the EuIch hybridization. Genetic analysis enabled us to unequivocally identify the hybrid origins of all triploid plants, except for one plant from the EuFor sexual hybridization and four from the EuIch sexual hybridization, which were rejected since they could have originated from autopollination of the female parents. Genetic analysis showed that “Eureka Frost” lemon produced the 2n gametes for all triploid hybrids, as shown in Figure 1.

For the tetraploid hybrids, the JK-TAA41 SSR marker displayed total allelic differentiation between “Fino” lemon and tetraploid *C. macrophylla*, allowing us to conclude that all plants were hybrids and that “Fino” lemon produced the 2n gametes (Figure 1). Analysis of the genetic origins of the 23 triploid plants recovered from this 2x X 4x hybridization showed that, as expected, they were obtained from the union of a normal reduced haploid female gamete and a normal reduced diploid pollen gamete, as previously observed in other citrus species (Aleza et al., 2012a).

Lemon hybrids were obtained from 2n gametes at a frequency of 4.9% for “Eureka Frost” and 8.3% for “Fino”. Geraci et al. (1975) reported frequencies of 1 and 5% for triploid hybrids assumed to be obtained through unreduced gametes of “Lisbon” and “Eureka” lemons, respectively. Pérez-Tornero et al. (2012) obtained triploid hybrids at a frequency of 5.8 to 8.6% in hybridizations between diploid plants of “Verna” as the female parent and “Fino” as the male parent. In mandarins, greater differences between genotypes have been observed, ranging from <1% for clementines to over 22% for “Sukega” and “Ortanique” tangor (Esen and Soost, 1971; Wakana et al., 1982; Ollitrault et al., 2008; Aleza et al., 2010a; Xie et al., 2014).

The frequency of 2n gametes was shown to be genotype-dependent in citrus and in other herbaceous and woody plants such as *Brassica*, potato, and peach (Dermen, 1938; Mok et al., 1975; Ollitrault et al., 2008; Aleza et al., 2010a; Mason et al., 2011; Younis et al., 2014). This hypothesis is supported by the genetic improvement of unreduced gamete rates for Trifolium (frequencies increased from 0.04 to 47%) and *Medicago sativa* (from 9 to 78%) in only three generations of recurrent selection (Gallais, 2003).

In the current study, we observed a rate of 4.9% 2n gametes in the 2x X 2x hybridizations (EuFor and EuIch), whereas, in the 2x X 4x hybridization (FinMac), the percentage was higher (8.3%). These differences might be due to a genotypic effect of the parents, but are more likely due to the modification of the embryo/endosperm ploidy level ratio in interploid hybridizations. Esen and Soost (1971) reported that, in diploid plants, when an unreduced gamete is pollinated with normal reduced pollen, the embryo/endosperm ploidy level ratio (3/5) is less favorable for embryo development than that for normal diploid embryos (2/3), whereas the pollination of a 2n female gamete with diploid pollen in 2x X 4x sexual hybridizations provides the correct embryo/endosperm ploidy level ratio (4/6 = 2/3), leading to normal seed development. Therefore, 2x X 4x hybridization appears to be a more favorable situation for revealing unreduced gametes via the development of tetraploid embryos in normal seeds.

### Mechanism of unreduced gamete formation

To determine the mechanism leading to unreduced gamete formation, we used nine unlinked molecular markers localized in the nine LGs for EuFor and EuIch and seven markers in seven different LGs for FinMac to perform a LOD score test for SDR/FDR and SDR/PRD probability ratios for all genotypes analyzed (Tables 3, 4, 5). The analysis of six markers covering LG1 and additional telomeric loci allowed us to distinguish between SDR and PMD when the inferred gametes were totally homozygous for the centromeric loci.

**Table 3:** Heterozygous and homozygous profiles for 2n gametes from EuFor hybridization analyzed using SSR and SNP markers close to the centromere of each LG and the LOD score test for SDR/FDR and SDR/PRD probability ratio.

**MARKER**	**MEST001**	**JK-CAC15**	**3p 11355960**	**mCrCIR07D06**	**5p 22687304**	**6p 7496245**	**mCrCIR03B07**	**8p 18684429**	**Ci07C09**	**LOD (SDR/ FDR)**	**LOD (SDR/PRD)**
**LG**	**1**	**2**	**3**	**4**	**5**	**6**	**7**	**8**	**9**		
Centromere Position (cM)	0.607	0.569	0.906	0.161	0.231	0.064	0.964	0.542	0.522		
Marker Position (cM)	0.706	0.435	0.885	0.163	0.210	0.065	0.834	0.560	0.530		
Distance to the centromere (cM)	0.099	0.134	0.021	0.002	0.021	0.001	0.130	0.018	0.008		
**Genotypes analyzed**	**2 n gamete genetic configuration**										
EuFor 1	HO	HO	HO	HO	HO	HO	HO	HO	HO	15.22	3.87
EuFor 2	HO	HO	HO	HO	HO	HO	HO	HO	HO	15.22	3.87
EuFor 3	HO	HO	HO	HO	HO	HO	HO	HO	HO	15.22	3.87
EuFor 4	HO	HO	HO	HO	HO	HO	HO	HO	HO	15.22	3.87
EuFor 5	HO	HO	HO	HO	HO	HO	HO	HE	HO	12.05	2.14
EuFor 6	HE	HO	HO	HO	HO	HO	HO	HO	HO	13.66	2.97
EuFor 7	HO	HO	HO	HO	HO	HO	HO	HO	HO	15.22	3.87
EuFor 8	HE	HO	HO	HO	HO	HO	HO	HO	HO	13.66	2.97
EuFor 9	HO	HO	HE	HO	HO	HO	HO	HO	HO	12.19	2.21
EuFor 10	HO	HO	HO	HO	HO	HO	HO	HO	HO	15.22	3.87
EuFor 11	HO	HE	HO	HO	HO	HO	HO	HO	HO	13.97	3.13
EuFor 12	HO	HE	HO	HO	HO	HO	HO	HO	HO	13.97	3.13
EuFor 13	HO	HO	HO	HO	HO	HO	HO	HO	HO	15.22	3.87
EuFor 14	HO	HO	HO	HO	HO	HO	HO	HO	HO	15.22	3.87
EuFor 15	HO	HO	HO	HO	HO	HO	HO	HO	HO	15.22	3.87
EuFor 16	HO	HE	HO	HO	HO	HO	HO	HO	HO	13.97	3.13
EuFor 17	HO	HE	HO	HO	HO	HO	HE	HO	HO	12.70	2.38
EuFor 18	HO	HE	HO	HO	HO	HO	HO	HO	HO	13.97	3.13
EuFor 19	HO	HO	HO	HO	HO	HO	HO	HO	HO	15.22	3.87
EuFor 20	HE	HO	HE	HE	HE	HO	HO	HE	HE	−4.52	−6.86
Population LODs										267.82	57.03

LODs >2 are significant for SDR. LOD < −2 are significant for FDR or PRD. LODs between 2 and −2 are not significant. HE: Heterozygous; HO: homozygous

**Table 4:** Heterozygous and homozygous profiles for 2n gametes from EuIch hybridization analyzed using SSR and SNP markers close to the centromere of each LG and the LOD score test for SDR/FDR and SDR/PRD probability ratio.

**MARKER**	**MEST001**	**JK-CAC15**	**3p11355960**	**mCrCIR07D06**	**5p22687304**	**CiC4356-06**	**mCrCIR03B07**	**8p18684429**	**9p4699283**	**LOD (SDR/FDR)**	**LOD (SDR/PRD)**
**LG**	**1**	**2**	**3**	**4**	**5**	**6**	**7**	**8**	**9**		
Centromere Position (cM)	0.607	0.569	0.906	0.161	0.231	0.064	0.964	0.542	0.522		
Marker Position (cM)	0.706	0.435	0.885	0.163	0.210	0.062	0.834	0.560	0.500		
Distance to the centromere (cM)	0.099	0.134	0.021	0.002	0.021	0.002	0.130	0.018	0.022		
**Genotypes analyzed**	**2 n gamete genetic configuration**										
EuIch 1	HE	HO	HO	HO	HO	HO	HE	HO	HE	8.69	0.55
EuIch 2	HO	HE	HO	HO	HO	HO	HO	HO	HO	13.28	3.12
EuIch 3	HO	HO	HO	HO	HO	HO	HO	HO	HO	14.53	3.86
EuIch 4	HO	HO	HO	HO	HO	HO	HO	HO	HE	11.53	2.21
EuIch 5	HO	HO	HO	HO	HO	HO	HO	HE	HO	11.36	2.13
EuIch 6	HE	HE	HO	HO	HO	HO	HO	HO	HO	11.72	2.21
EuIch 7	HO	HO	HO	HO	HO	HO	HO	HO	HO	14.53	3.86
EuIch 8	HE	HO	HO	HO	HO	HO	HO	HO	HO	12.97	2.95
EuIch 9	HE	HE	HE	HE	HE	HE	HO	HO	HE	−7.60	−8.19
EuIch 10	HE	HO	HE	HE	HE	HE	HE	HE	HE	−10.80	−9.93
Population LODs										80.21	2.77

*LODs > 2 are significant for SDR. LOD < −2 are significant for FDR or PRD. LODs between 2 and −2 are not significant. HE, Heterozygous; HO, Homozygous*.

**Table 5:** Heterozygous and homozygous profiles for 2n gametes from FinMac hybridization analyzed using SSR and SNP markers close to the centromeres of seven LGs and the LOD score test for SDR/FDR and SDR/PRD probability ratio.

**MARKER**	**MEST001**		**JK-CAC15**	**CiC4240-04**	**LapXcF238**	**mCrCIR03B07**	**8P16570424**	**CiBE3966**	**LOD (SDR/FDR)**	**LOD (SDR/PRD)**
**LG**	**1**		**2**	**4**	**6**	**7**	**8**	**9**		
Centromere Position (cM)	0.607		0.569	0.161	0.064	0.964	0.542	0.522		
Marker Position (cM)	0.706		0.435	0.071	0.110	0.834	0.500	0.523		
Distance to the centromere (cM)	0.099		0.134	0.091	0.046	0.130	0.042	0.001		
**Genotypes analyzed**	**2n gamete genetic configuration**									
FinMac 1	HO	HO		HO	HO	HO	HO	HO	8.93	2.81
FinMac 2	HO	HO		HO	HO	HO	HO	HO	8.93	2.81
FinMac 3	HO	HO		HO	HO	HO	HO	HO	8.93	2.81
FinMac 4	HO	HO		HO	HO	HO	HO	HO	8.93	2.81
FinMac 5	HO	HO		HO	HE	HO	HO	HO	6.62	1.52
FinMac 6	HE	HO		HO	HO	HO	HO	HO	7.37	1.91
FinMac 7	HO	HO		HE	HO	HO	HE	HO	4.88	0.52
FinMac 8	HO	HO		HE	HO	HO	HO	HO	7.28	1.86
FinMac 9	HO	HO		HE	HE	HO	HO	HO	4.97	0.56
FinMac 10	HO	HO		HE	HO	HE	HO	HO	6.00	1.10
FinMac 11	HO	HO		HE	HO	HE	HO	HO	6.00	1.10
Population LODs									78.84	19.81

*LODs > 2 are significant for SDR. LOD < −2 are significant for FDR or PRD. LODs between 2 and −2 are not significant. HE: Heterozygous and HO Homozygous*.

### LOD score analysis

For the EuFor hybridization, 20 triploid hybrids were genotyped using nine centromeric loci found in all LGs. Ten of the inferred 2n gametes were totally homozygous for these markers. However, all displayed at least one heterozygous marker when six markers covering LG1 were analyzed, allowing the PMD hypothesis to be rejected for all inferred 2n gametes. For the SDR/FDR hypothesis test at the individual level, 19 inferred 2n gametes displayed LOD values >2 (ranging from 12.05 to 15.22; Table 3). For the same 19 gametes, the LOD values for SDR/PRD were also >2. Therefore, these 19 plants were considered to have originated from SDR. One plant obtained negative LODs of −4.52 and −6.86 for the SDR/FDR and SDR/PRD hypotheses, respectively, suggesting that this plant is of FDR or PRD origin. At the population level, the LOD values were 267.82 and 57.03 for the SDR/FDR and SDR/PRD hypotheses, respectively, revealing a high rate of SDR.

For EuIch hybridization, 10 triploid hybrids were genotyped with nine centromeric markers located on all LGs. Two inferred 2n gametes were totally homozygous for these markers, but at least one heterozygous locus was observed for each 2n gamete in the complementary analysis of PHR along the LG1, thus discarding the PMD hypothesis. At the individual level, eight plants displayed LOD values >2 for SDR/FDR (from 8.69 to 14.53), rejecting the FDR hypothesis (Table 4). Among them, seven displayed a LOD >2 for SDR/PRD (ranging from 2.13 to 3.86) and were considered to have arisen from SDR. The LOD value for the remaining 2n gamete was 0.55, suggesting that this 2n gamete had arisen from SDR rather than PRD, but, since this value is below our threshold, this result is not conclusive. Two plants produced negative LOD values (< −2) in both the SDR/FDR and SDR/PRD tests, suggesting that they originated by FDR or PRD. The population LODs were 80.21 and 2.77 for SDR/FDR and SDR/PRD respectively, confirming the predominance of the SDR mechanism.

For FinMac, 13 tetraploid hybrids were genotyped with seven centromeric markers (LGs 1, 2, 4, 6, 7, 8, and 9). Six inferred 2n gametes were totally homozygous for these markers (Table 5). Among these, two unreduced gametes (from FinMac 12 and FinMac 13) remained totally homozygous after analyzing six markers covering LG1 and were subjected to additional analysis to distinguish between the SDR and PMD hypothesis. The 11 2n gametes with at least one heterozygous locus produced LOD values >2 for SDR/FDR, rejecting the FDR hypothesis. Among these, four displayed LOD values of 2.81 for the SDR/PRD test and were therefore considered to have arisen from SDR. The seven remaining 2n gametes displayed positive values ranging from 0.52 to 1.91. These gametes had a higher probability of arising from SDR than from PRD, but this result is not conclusive because the values are below our threshold. The population LOD values were 78.84 and 19.81 for SDR/FDR and SDR/PRD, respectively, again confirming the prevalence of SDR. The seven 2n gametes with inconclusive individual LODs display a population LOD of 43.12 and 8.56 for SDR/FDR and SDR/PRD, respectively. It is therefore highly probable that they also arose from SDR.

### Pattern of heterozygosity restitution along Lg1 for 2n gametes with an identified SDR origin and undetermined SDR/PRD origin

**Figure 2:**
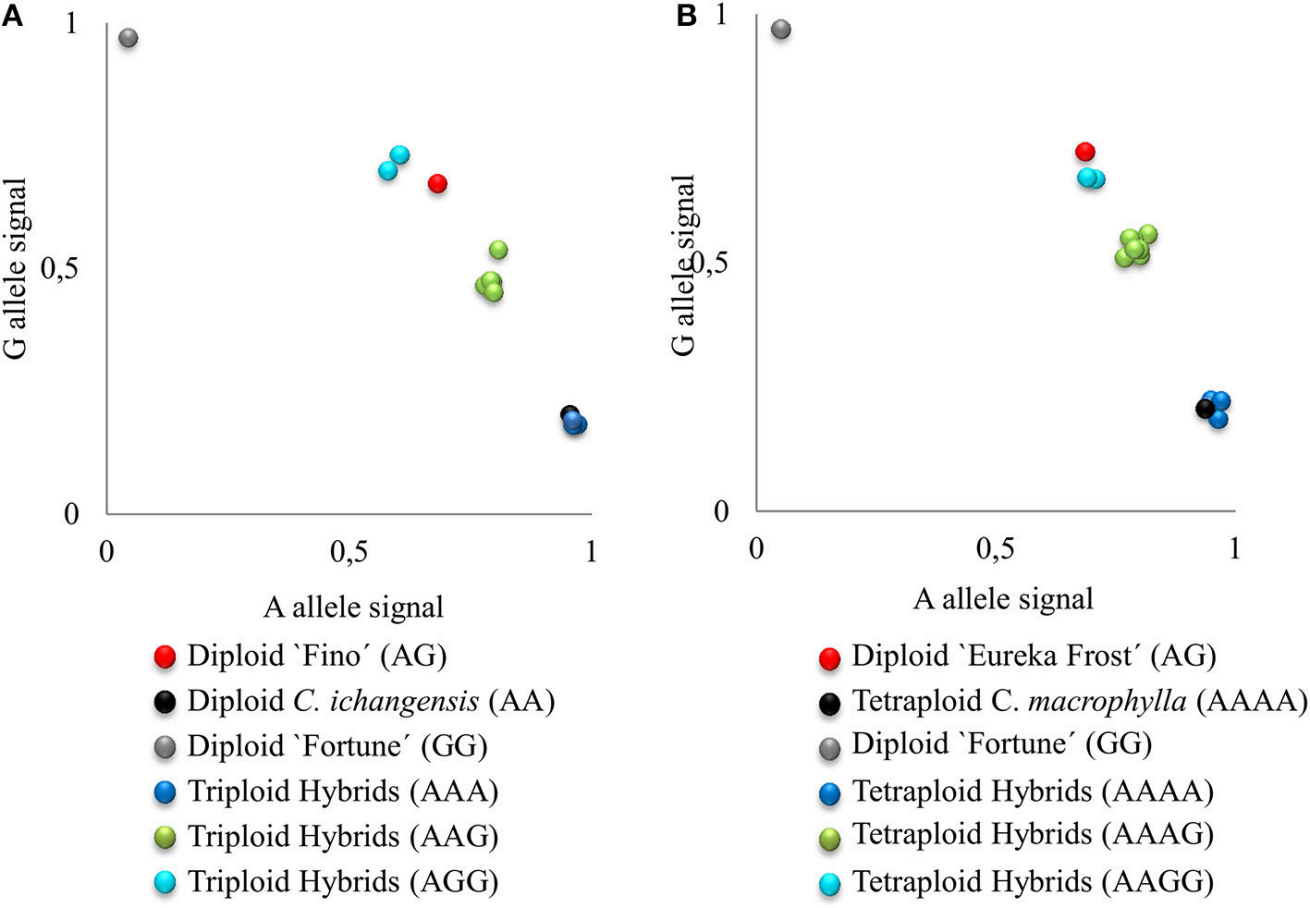
Plot of A, G allele signals of SNP marker CiC5950-02 representing triploid **(A)** and tetraploid **(B)** hybrids from EuIch and FinMac sexual hybridizations. Letters indicate the allelic configuration for each hybrid.

**Figure 3:**
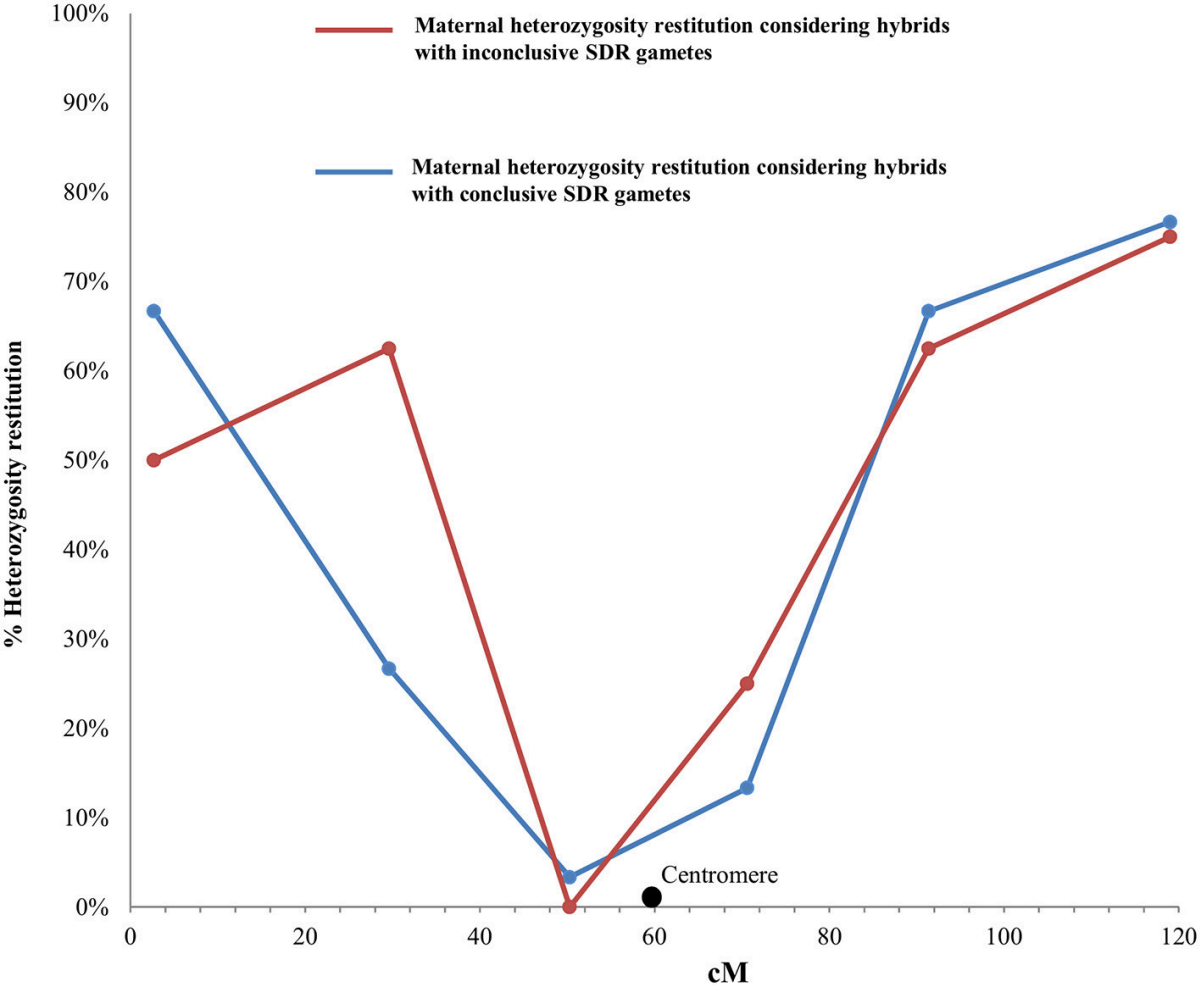
Evolution of maternal heterozygosity restitution values of the analyzed SSR and SNP markers in LG1 considering the significance of the obtained LOD values for each hybrid from “Eureka Frost” and “Fino” lemons with conclusive and inconclusive SDR2n gametes. Black dot indicates the centromere position on the reference clementine genetic map (Ollitrault et al., 2012a).

**Figure 4:**
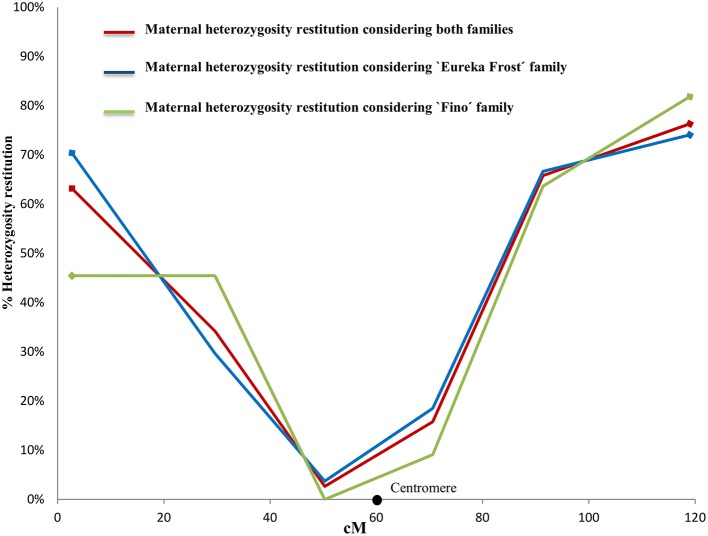
Evolution of maternal heterozygosity restitution values of the analyzed SSR and SNP markers in LG 1 considering both populations, “Eureka Frost” and “Fino” lemon SDR-2n gametes. Black dots indicate the centromere position on the reference clementine genetic map (Ollitrault et al., 2012a).

To validate, at the population level, the finding that 38 2n gametes were derived by SDR (as determined by individual LOD analysis) and to distinguish between SDR and PRD for the eight gametes with inconclusive individual LODs, we compared the PHR patterns of the two set of gametes in LG1. For this analysis, we used four SSR markers (CIBE6126, mCrCIR06B05, MEST001, and MEST431) and two SNP markers (CiC2110-02 and CiC5950-02) (Figure 2) mapped in LG1 (Figures 3, 4).

For the conclusive SDR 2n gametes, the PHR values in LG1 (Figure 3) decreased from 67% for the telomeric marker CIBE6126 to 3% for the centromeric marker mCrCIR06B05 and progressively increased to 77% when moving toward the other telomeric marker, MEST431. The average PHR value was 42%. For the eight inconclusive 2n gametes, the same PHR pattern was observed: the lowest value was obtained for the centromeric marker mCrCIR06B05 (0%) and the highest for the telomeric markers (63% for CiC2110-02 in one telomere and 75% for MEST431 in the other). The average PHR for these eight gametes was 46% (Figure 3). These PHR patterns totally fit the profile for SDR. The average PHR value over the two sets of 2n gametes was 43%. Various studies have indicated that the global restitution of heterozygosity is expected to be near 80% for FDR and 40% for SDR, assuming a random distribution of heterozygous loci along the chromosomes (Peloquin, 1983; Hutten et al., 1994; Carputo et al., 2003). Both the patterns along LG1 and the average PHR values comply with the SDR hypothesis. Therefore, we conclude that the eight 2n gametes of indeterminate origin identified from the individual LOD (SDR/PRD) analysis also originated from SDR. Under this conclusion, the PHR pattern in LG 1 is very similar for “Eureka Frost” and “Fino” lemon SDR 2n gamete populations (Figure 4).

### Distinction between SDR and PMD for fully homozygous 2n gametes

**Figure 5:**
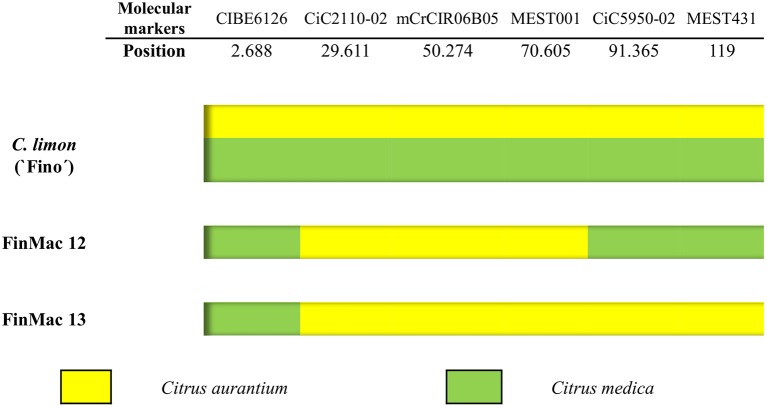
Multilocus configuration of the two fully homozygous plants recovered from FinMac hybridization with six molecular markers located on LG 1. Yellow indicates the presence of homozygous alleles inherited from *C. aurantium*, and green indicates those from *C. medica*.

We performed additional analyses of the two inferred 2n gametes (FinMac 12 and FinMac 13 tetraploid plants) fully homozygous for the seven centromeric markers and the six markers of LG1. Fully homozygous 2n female gametes for centromeric loci can originate through SDR or PMD, with different consequences for the genetic structures of 2n gametes. Bastiaanssen et al. (1998) defined two conditions that are necessary to conclude that PMD rather than SDR has occurred, i.e., 100% homozygosity for all genotyped loci and the occurrence of recombination between homozygous alleles in the same LG. Therefore, we genotyped FinMac 12 and FinMac 13 using 11 telomeric loci found in different LGs to provide genetic evidence for a particular PMD mechanism. The average distance from these markers to their corresponding centromere is 53.22 cM (ranging from 25.32 to 89.59 cM). Both plants were homozygous for all molecular markers analyzed. Furthermore, *C. limon* is a direct hybrid between two genetically distant genotypes, *C. aurantium* and *C. medica* (Nicolosi et al., 2000; Curk et al., 2016), and the specific origins of the homozygous alleles can easily be distinguished. We found that some homozygous markers of the same LG were inherited from the *C. aurantium* ancestor and the others from *C. medica*. For example, multilocus analyses of the homozygous alleles in LG1 (Figure 5) revealed interspecific recombination in the two plants with alternation of homozygosity originated from *C. aurantium* and *C. medica*. Consequently, according to Bastiaanssen et al. (1998), the observation of 100% homozygosity and recombination between *C. aurantium* and *C. medica* along the same LG provides evidence discarding the SDR mechanism and leads us to conclude that these two 2n gametes originated through PMD. To our knowledge, this is the first report of the identification of a new mechanism, Post-Meiotic genome Doubling, leading to 2n ovule gametes in citrus, and specifically in lemon.

### Synthesis of different approaches

On the whole, we conclude that 38 (88%) of the 2n gametes analyzed had arisen from SDR, three (7%) from FDR or PRD, and two (5%) from PMD. At the population level, SDR appears to be by far the most common mechanism for 2n ovule formation in both *C. limon* genotypes, “Eureka Frost” and “Fino”. This is the first report of the production of a large number of lemon progenies from 2n gametes produced by different mechanisms of unreduced ovule gametes. Luro et al. (2004), Aleza et al. (2015), and Cuenca et al. (2015) also found that SDR was the predominant mechanism leading to 2n megagametophyte production in mandarins. Among the 19 mandarins investigated, the authors concluded that only 1.1 and 2.9% of plants were recovered from FDR in the “Ellendale” and “Fortune” mandarins, respectively. The coexistence of SDR and FDR has been recently observed in unreduced pollen gametes by Rouiss et al. (2017). 53 plants were obtained from 2n pollen gametes produced by a diploid hybrid between clementine and sweet orange. FDR was the predominant mechanism (77%) and SDR was the mechanism for the remaining plants (23%). In addition, FDR was the main mechanism for 2n female gamete production in “Femminello” lemon (Ferrante et al., 2010). These results are questionable because the authors used only a few molecular markers and lacked previous information about centromere location and the relative distances between the markers and the centromeres. With the recent location of centromeres in the citrus genetic map (Ollitrault et al., 2012a; Aleza et al., 2015), the markers used by Ferrante et al. (2010), JK-TAA1, JK-TAA15, JK-TAA41, and NB-GT03, are located at 87.29, 59.07, 74.99, and 50.47 cM from the centromere of the LGs 6, 1, 2, and 8 respectively, being mostly telomeric, and therefore the high PHR values obtained in their study can fit both SDR or FDR mechanisms.

At the methodological level, we demonstrated the power of using two complementary approaches, namely, analysis of the PHR pattern in one LG with the maximum-likelihood method proposed by Cuenca et al. (2015). Considering only centromeric loci, different mechanisms can lead to the same homozygous patterns. Therefore, analyzing the heterozygosity restitution pattern along LGs at the individual level is a useful approach for distinguishing between SDR and PMD, since, under this mechanism, the heterozygosity restitution value is zero for all markers in all LGs. After LOD analysis at the individual level, this method is used to analyze PHR patterns at the population level to distinguish between SDR and PRD when individual LODs are under the threshold required to obtain conclusive results. When enough number of individuals is analyzed, this technique should also be utilized to distinguish between FDR and PRD. With FDR-2n gametes, heterozygosity restitution varies from 100% in centromeric loci to close to 66% in telomeric areas under the non-interference model (Cuenca et al., 2011), whereas, with PRD, heterozygosity restitution is expected to be very similar along the entire chromosome.

### Crossover and interference analysis

Crossover interference ensures the appropriate distribution of crossovers along the chromosome, since one crossover reduces the likelihood of other crossovers occurring nearby (Youds et al., 2010). The analysis of crossover rates (Table 6) for both arms of chromosome I revealed the presence of up to four crossovers on one arm and three on the other arm. In addition, three complementary crossovers (double crossing over involving four chromatids) were observed as a result of phase-changing between two homozygous markers. Similarly, Cuenca et al. (2011) and Aleza et al. (2015) detected up to four crossovers on one arm and complementary crossovers in “Fortune” mandarin and *C. clementina*. We estimated the IC for each chromosome arm, finding partial interference in both arms (IC = 0.27 and 0.44). Such variation in interference values between both arms has also been observed in other citrus species, ranging from 0.82 to 0.48 for “Fina” clementine on LG 1 (Aleza et al., 2015) and 0.73 to 0.53 for “Fortune” mandarin on LG 2 (Cuenca et al., 2011). Variation in the level of interference between different parts of the genome has also been observed in Arabidopsis (Drouaud et al., 2007), humans (Lian et al., 2008), and mice (Broman et al., 2002).

**Table 6:** Number of observed crossover events on each arm of chromosome I based on analysis of 27 genotypes recovered from “Eureka Frost” lemon pollinated with *C. ichangensis* and “Fortune” mandarin using six molecular markers.

**Number of crossovers**		**Arm 1**					
		**0**	**1**	**2**	**3**	**4**	
**Arm 2**	0	2	2	1	0	0	13%
	1	7	17	3 (2)	0	1(1)	74%
	2	1	3	0	0	0	11%
	3	0	1	0	0	0	3%
		26%	61%	11%	0%	3%	

*Numbers between brackets indicate the number of complementary crossovers*.

### Implications of sexual polyploidization for breeding triploid lemon-like plants

Sexual polyploidization via 2n gametes and interploid sexual hybridizations using tetraploid parents (doubled diploids) are the main strategies used to produce triploid citrus hybrids (Ollitrault et al., 2008; Aleza et al., 2010b, 2012a,b, 2016; Navarro et al., 2015). These different strategies and the different meiotic behaviors result in different genetic structures in the diploid gametes and, consequently, the resulting triploid progenies. The three hybrids obtained via FDR or PRD 2n gametes have a higher rate of heterozygosity than hybrids obtained via SDR. By contrast, the two plants obtained by PMD transmit 0% of PHR (Bastiaanssen et al., 1998). Therefore, such a mechanism generally promotes inbreeding in the hybrid progenies (Tai, 1986; Gallais, 2003). However, these lines constitute interesting parentals to be used as test lines in inheritance studies (Bastiaanssen et al., 1998).

In addition, the mechanism that generates the 2n gametes affects the breeding efficiency for a character in relation to the genetic distance to the centromeres of the major genes controlling this character. For instance, Cuenca et al. (2013b, 2016) found that resistance to Alternaria brown-spot fungal disease is a recessive trait controlled by a single locus located at 10.5 cM from the centromere of chromosome III. Therefore, in crosses between a heterozygous parent producing diploid gametes and a resistant genotype, PMD is the most favorable mechanism (50% of resistant hybrids), followed by SDR (40%). Under FDR, only 5% of the hybrids will be resistant. For diploid gametes produced by the doubled-diploid genotype or resulting from PRD, the rates of resistant hybrids should vary from 16% (tetrasomic segregation) to 0% (disomic segregation) according to the preferential pairing behavior.

The aim of some lemon-breeding programs is to produce new lemon-like types of fruit, which essentially involves 2x X 4x crosses using diploid lemons as female parents and more or less complex hybrids as tetraploid parents (Recupero et al., 2005; Viloria and Grosser, 2005). This approach is used in an attempt to solve some of the problems caused by the low genetic variation of *C. limon*, although relatively few tetraploids are available. This approach has allowed for the selection and protection of the triploid “Lemox”, a hybrid between a diploid female complex hybrid, and tetraploid lemon (Recupero et al., 2005). “Lemox” produces quality fruits resembling lemons with high tolerance to Mal secco. The 2n lemon gametes will be very useful for producing new lemon-like seedless citrus types via 2x X 2x hybridizations, thereby dramatically increasing the gene pool of genotypes that could be used as parents. Furthermore, the production of 2n gametes has been investigated in a small number of lemon genotypes. Evaluating the many existing lemon genotypes may result in the detection of specific genotypes that produce higher rates of 2n gametes and (eventually) genotypes with different ratios of FDR and SDR 2n gametes, which will increase the efficiency of breeding programs.

## Conclusion

Genetic analysis with SSR and SNP markers revealed that two genotypes of *C. limon*, “Eureka Frost” and “Fino”, produced 2n female gametes. The frequencies of 2n gametes were 4.9 and 8.3% for “Eureka Frost” and “Fino” lemons, respectively. The use of complementary methods, including individual LOD analysis from centromeric loci, telomeric loci genotyping, and the analysis of PHR patterns along a LG, allowed us to distinguish among the different mechanisms of 2n gamete formation. We detected three meiotic mechanisms in lemon, with 88% of 2n female gametes arising from SDR, 7% from FDR or PRD, and 5% from PMD. To our knowledge, this is the first report of the production of a large number of lemon progenies from 2n gametes and the identification of a new mechanism, PMD, which had never been observed in citrus and rarely been described in other herbaceous or woody species. From the breeding point of view, the production of SDR-2n gametes would allow progenies with polymorphic genetic structures to be recovered, increasing the likelihood of obtaining new phenotypes by creating an increasing number of novel multilocus allelic combinations. The coexistence of different mechanisms for 2n gamete formation broadens the diversity of lemon 2n gametes and, therefore, their potential for breeding.

## Author contributions

LN, PO, and PA conceived and designed the experiments. HR performed the experiments. HR and PA analyzed the data. JC and PO provided a statistical method for the estimation of SDR and FDR mechanisms. HR, PA, LN, and PO wrote the manuscript.

### Conflict of interest statement

The authors declare that the research was conducted in the absence of any commercial or financial relationships that could be construed as a potential conflict of interest.

## References

[B1] Aleza P. Cuenca J. Hernández M. Juárez J. Navarro L. Ollitrault P. (2015). Genetic mapping of centromeres of the nine Citrus clementina chromosomes using half-tetrad analysis and recombination patterns in unreduced and haploid gametes. BMC Plant Biol. 15:80. 10.1186/s12870-015-0464-y 25848689 PMC4367916

[B2] Aleza P. Cuenca J. Juárez J. Navarro L. Ollitrault P. (2016). Inheritance in doubled-diploid clementine and comparative study with SDR unreduced gametes of diploid clementine. Plant Cell Rep. 35, 1573–1586. 10.1007/s00299-016-1972-4 27038940

[B3] Aleza P. Juárez J. Cuenca J. Ollitrault P. Navarro L. (2010a). Recovery of citrus triploid hybrids by embryo rescue and flow cytometry from 2x × 2x sexual hybridisation and its application to extensive breeding programs. Plant Cell Rep. 29 1023–1034. 10.1007/s00299-010-0888-7 20607244

[B4] Aleza P. Juárez J. Cuenca J. Ollitrault P. Navarro L. (2012a). Extensive citrus triploid hybrid production by 2x× 4x sexual hybridizations and parent-effect on the length of the juvenile phase. Plant Cell Rep. 31, 1723–1735. 10.1007/s00299-012-1286-0 22614256

[B5] Aleza P. Juárez J. Hernández M. Ollitrault P. Navarro L. (2012b). Implementation of extensive citrus triploid breeding programs based on 4x× 2x sexual hybridisations. Tree Genet. Genomes 8, 1293–1306. 10.1007/s11295-012-0515-6

[B6] Aleza P. Juárez J. Ollitrault P. Navarro L. (2010b). Polyembryony in non-apomictic citrus genotypes. Ann. Bot. 106, 533–545. 10.1093/aob/mcq148 20675656 PMC2944972

[B7] Bastiaanssen H. J. Van Den Berg Petra M. M. M. Lindhout P. Jacobsen E. Ramanna M. (1998). Postmeiotic restitution in 2n-egg formation of diploid potato. Heredity 81, 20–27. 10.1046/j.1365-2540.1998.00370.x

[B8] Broman K. W. Rowe L. B. Churchill G. A. Paigen K. (2002). Crossover interference in the mouse. Genetics 160, 1123–1131. Available online at: https://www.biostat.wisc.edu/~kbroman/publications/mousebc.pdf 11901128 10.1093/genetics/160.3.1123 PMC1462020

[B9] Calabrese F. De Michele A. Barone F. (2000). New seedless lemon varieties for Sicily, in Programs & Abstracts. (P129). IXth Congress of the International Society of Citriculture, 114 (Orlando, FL).

[B10] Cameron J. Burnett R. (1978). Use of sexual tetraploid seed parents for production of triploid Citrus hybrids. HortScience 13, 167–169.

[B11] Carputo D. Frusciante L. Peloquin S. J. (2003). The role of 2n gametes and endosperm balance number in the origin and evolution of polyploids in the tuber-bearing Solanums. Genetics 163, 287–294. Available online at: http://www.genetics.org/content/genetics/163/1/287.full.pdf 12586716 10.1093/genetics/163.1.287 PMC1462417

[B12] Cuenca J. Aleza P. Garcia-Lor A. Ollitrault P. Navarro L. (2016). Fine Mapping for Identification of Citrus Alternaria Brown Spot Candidate Resistance Genes and Development of New SNP Markers for Marker-Assisted Selection. Front. Plant Sci. 7:1948. 10.3389/fpls.2016.01948 28066498 PMC5179576

[B13] Cuenca J. Aleza P. Juárez J. García-Lor A. Froelicher Y. Navarro L.. (2015). Maximum-likelihood method identifies meiotic restitution mechanism from heterozygosity transmission of centromeric *loci*, application in citrus. Sci. Rep. 5:9897. 10.1038/srep09897 25894579 PMC4403285

[B14] Cuenca J. Aleza P. Navarro L. Ollitrault P. (2013a). Assignment of SNP allelic configuration in polyploids using competitive allele-specific PCR, application to citrus triploid progeny. Ann. Bot. 111, 731–742. 10.1093/aob/mct032 23422023 PMC3605964

[B15] Cuenca J. Aleza P. Vicent A. Brunel D. Ollitrault P. Navarro L. (2013b). Genetically based location from triploid populations and gene ontology of a 3.3-Mb genome region linked to alternaria brown spot resistance in citrus reveal clusters of resistance genes. PloS ONE 8:e76755. 10.1371/journal.pone.0076755 24116149 PMC3792864

[B16] Cuenca J. Froelicher Y. Aleza P. Juárez J. Navarro L. Ollitrault P. (2011). Multilocus half-tetrad analysis and centromere mapping in citrus, evidence of SDR mechanism for 2n megagametophyte production and partial chiasma interference in mandarin cv ‘Fortune’. Heredity 107, 462–470. 10.1038/hdy.2011.33 21587302 PMC3199928

[B17] Cuppen E. (2007). Genotyping by Allele-Specific Amplification. (KASPar). CSH Protoc. 2007. 10.1101/pdb.prot4841 21357174

[B18] Curk F. Ancillo G. Ollitrault F. Perrier X. Jacquemoud-Collet J. P. García-Lor A.. (2015). Nuclear species-diagnostic SNP markers mined from 454 amplicon sequencing reveal admixture genomic structure of modern Citrus varieties. PloS ONE 10:e0125628. 10.1371/journal.pone.0125628 25973611 PMC4431842

[B19] Curk F. Ollitrault F. García-Lor A. Luro F. Navarro L. Ollitrault P. (2016). Phylogenetic origin of limes and lemons revealed by cytoplasmic and nuclear markers. Ann. Bot. 117, 565–583. 10.1093/aob/mcw005 26944784 PMC4817432

[B20] De Storme N. Geelen D. (2013). Sexual polyploidization in plants–cytological mechanisms and molecular regulation. New Phytol. 198, 670–684. 10.1111/nph.12184 23421646 PMC3744767

[B21] Dermen H. (1938). Detection of polyploidy by grain size, investigation with peaches and apricots. J. Am. Soc. Hortic Sci. 35, 96–103.

[B22] Drouaud J. Mercier R. Chelysheva L. Berard A. Falque M. Martin O.. (2007). Sex-specific crossover distributions and variations in interference level along *Arabidopsis thaliana* chromosome 4. PLoS Genet. 3:106. 10.1371/journal.pgen.0030106 17604455 PMC1904369

[B23] Duportal M. Jorda E. Sanchez C. Imbert É. Loeillet D. Vannière H. (2013). FruiTrop FOCUS Citron. FruiTrop Cirad 2013, 140 Available online at: http://www.fruitrop.com/media/Publications/FruiTrop-FOCUS

[B24] Esen A. Soost R. K. (1971). Unexpected triploids in Citrus, their origin, identification, and possible use. J. Hered. 62, 329–333. 10.1093/oxfordjournals.jhered.a108186

[B25] Esen A. Soost R. K. (1973a). Precocious development and germination of spontaneous triploid seeds in Citrus. J. Hered. 64, 147–154. 10.1093/oxfordjournals.jhered.a108373

[B26] Esen A. Soost R. K. (1973b). Seed development in Citrus with special reference to 2x x 4x crosses. Am. J. Bot. 60, 448–462.

[B27] Esen A. Soost R. K. (1975). Triploid progenies of Citrus cultivars from 2x× 2x crosses. J. Hered. 66, 177–178. 10.1093/oxfordjournals.jhered.a1086071172514

[B28] Esselink G. Nybom H. Vosman B. (2004). Assignment of allelic configuration in polyploids using the MAC-PR (microsatellite DNA allele counting—peak ratios). method. Theor. Appl. Genet. 109, 402–408. 10.1007/s00122-004-1645-5 15085263

[B29] Ferrante S. P. Lucretti S. Reale S. De Patrizio A. Abbate L. Tusa N. (2010). Assessment of the origin of new citrus tetraploid hybrids. (2n = 4x). by means of SSR markers and PCR based dosage effects. Euphytica 173, 223–233. 10.1007/s10681-009-0093-3

[B30] Froelicher Y. Dambier D. Bassene J. Costantino G. Lotfy S. Didout C.. (2008). Characterization of microsatellite markers in mandarin orange. (Citrus reticulata Blanco). Mol. Ecol. Resour. 8, 119–122. 10.1111/j.1471-8286.2007.01893.x 21585732

[B31] Froelicher Y. Mouhaya W. Bassene J. Costantino G. Kamiri M. Luro F. (2011). New universal mitochondrial PCR markers reveal new information on maternal citrus phylogeny. Tree Genet. Genomes 7, 49–61. 10.1007/s11295-010-0314-x

[B32] Gallais A. (2003). Quantitative Genetics and Breeding Methods in Autopolyploid Plants. Paris: INRA Editions.

[B33] Garcia-Lor A. Curk F. Snoussi-Trifa H. Morillon R. Ancillo G. Luro F.. (2013). A nuclear phylogenetic analysis, SNPs, indels and SSRs deliver new insights into the relationships in the ‘true citrus fruit trees’ group. (Citrinae, Rutaceae). and the origin of cultivated species. Ann. Bot. 111, 1–19. 10.1093/aob/mcs227 23104641 PMC3523644

[B34] García-Lor A. Luro F. Navarro L. Ollitrault P. (2012). Comparative use of InDel and SSR markers in deciphering the interspecific structure of cultivated citrus genetic diversity, a perspective for genetic association studies. Mol. Genet. Genomics 287, 77–94. 10.1007/s00438-011-0658-4 22160318

[B35] Geraci G. Esen A. Soost R. (1975). Triploid progenies 2x X 2x crosses of Citrus cultivars. J. Hered. 66, 177–178. 1172514

[B36] Griffiths A. J. F. Miller J. H. Suzuki D. T. Lewontin R. C. Gelbart W. M. (1996). An Introduction to Genetic Analysis Ed 6W. New York, NY: H Freeman and Company.

[B37] Grosser J. W. An H. J. Calovic M. Lee D. H. Chen C. Vasconcellos M. (2010). Production of new allotetraploid and autotetraploid citrus breeding parents, focus on zipperskin mandarins. HortScience 45, 1160–1163. Available online at: http://hortsci.ashspublications.org/content/45/8/1160.full

[B38] Grosser J. W. Gmitter F. G. Jr. (2011). Protoplast fusion for production of tetraploids and triploids, applications for scion and rootstock breeding in citrus. Plant Cell Tissue Organ Cult. 104, 343–357. 10.1007/s11240-010-9823-4

[B39] Hutten R. Schippers M. Hermsen J. T. Ramanna M. (1994). Comparative performance of FDR and SDR progenies from reciprocal 4x-2x crosses in potato. Theor. Appl. Genet. 89, 545–550. 10.1007/BF00222446 24177928

[B40] Kijas J. M. H. Thomas M. R. Fowler J. C. S. Roose M. L. (1997). Integration of trinucleotide microsatellites into a linkage map of Citrus. Theor. Appl. Genet. 94, 701–706. 10.1007/s001220050468

[B41] Krueger R. R. Navarro L. (2007). Citrus germplasm resources, in Citrus Genetics, Breeding, and Biotechnology, ed Khan I. A. (Wallingford, CT: CAB International), 45–140.

[B42] Krug C. (1943). Chromosome numbers in the subfamily Aurantioideae with special reference to the genus Citrus. Bot. Gaz. 104, 602–611 10.1086/335173

[B43] Lee L. (1988). Citrus polyploidy-origins and potential for cultivar improvement. Crop. Pasture Sci. 39, 735–747. 10.1071/AR9880735

[B44] Lian J. Yin Y. Oliver-Bonet M. Liehr T. Ko E. Turek P.. (2008). Variation in crossover interference levels on individual chromosomes from human males. Hum. Mol. Genet. 17, 2583–2594. 10.1093/hmg/ddn158 18502786

[B45] Luro F. Costantino G. Terol J. Argout X. Allario T. Wincker P.. (2008). Transferability of the EST-SSRs developed on Nules clementine. (Citrus clementina Hort exTan). to other Citrus species and their effectiveness for genetic mapping. BMC Genomics 9:287. 10.1186/1471-2164-9-287 18558001 PMC2435559

[B46] Luro F. Maddy F. Jacquemond C. Froelicher Y. Morillon R. Rist D. (2004). Identification and evaluation of diplogyny in clementine. *(Citrus clementina)* for use in breeding. Acta Hortic. 663, 841–847. 10.17660/ActaHortic.2004.663.152

[B47] Martín J. J. H. González J. C. (2014). La fruticultura del siglo XXI en España. Cajamar Caja Rural Editions.

[B48] Mason A. S. Nelson M. N. Yan G. Cowling W. A. (2011). Production of viable male unreduced gametes in Brassica interspecific hybrids is genotype specific and stimulated by cold temperatures. BMC Plant Biol. 11:103. 10.1186/1471-2229-11-103 21663695 PMC3141635

[B49] Mendiburu A. O. Peloquin S. J. (1976). Sexual polyploidization and depolyploidization, some terminology and definitions. Theor. Appl. Genet. 48, 137–143. 10.1007/BF00281656 24413691

[B50] Migheli Q. Cacciola S. A. Balmas V. Pane A. Ezra D. di San Lio G. M. (2009). Mal Secco disease caused by *Phoma tracheiphila*, a potential threat to lemon production worldwide. Plant Dis. 93, 852–867. 10.1094/PDIS-93-9-0852 30754534

[B51] Mok D. Peloquin S. Tarn T. (1975). Cytology of potato triploids producing 2n pollen. Am. Potato J. 52, 171–174. 10.1007/BF02838107

[B52] Navarro L. Aleza P. Cuenca J. Juárez J. Pina J. A. Ortega C. (2015). The mandarin triploid breeding program in Spain. Acta Hortic 1065, 389–396. 10.17660/ActaHortic.2015.1065.48

[B53] Nicolosi E. Deng Z. Gentile A. La Malfa S. Continella G. Tribulato E. (2000). Citrus phylogeny and genetic origin of important species as investigated by molecular markers. Theor. Appl. Genet. 100, 1155–1166. 10.1007/s001220051419

[B54] Ollitrault F. Terol J. Pina J. A. Navarro L. Talon M. Ollitrault P. (2010). Development of SSR markers from Citrus clementina. (Rutaceae). BAC end sequences and interspecific transferability in Citrus. Am. J. Bot. 97, e124–e129. 10.3732/ajb.1000280 21616814

[B55] Ollitrault P. Dambier D. Luro F. Froelicher Y. (2008). Ploidy manipulation for breeding seedless triploid citrus. Plant Breed. Rev. 30, 323–352. 10.1002/9780470380130.ch7

[B56] Ollitrault P. Terol J. Chen C. Federici C. T. Lotfy S. Hippolyte I.. (2012a). A reference genetic map of C. clementina hort. ex Tan.; citrus evolution inferences from comparative mapping. BMC Genomics 13:593. 10.1186/1471-2164-13-593 23126659 PMC3546309

[B57] Ollitrault P. Terol J. García-Lor A. Berard A. Chauveau A. Froelicher Y.. (2012b). SNP mining in C. clementina BAC end sequences; transferability in the Citrus genus. (Rutaceae), phylogenetic inferences and perspectives for genetic mapping. BMC Genomics 13:13. 10.1186/1471-2164-13-13 22233093 PMC3320530

[B58] Park T. H. Kim J. B. Hutten R. C. van Eck H. J. Jacobsen E. Visser R. G. (2007). Genetic positioning of centromeres using half-tetrad analysis in a 4x-2x cross population of potato. Genetics 176, 85–94. 10.1534/genetics.107.070870 17339217 PMC1893073

[B59] Peloquin S. (1983). Genetic Engineering with Meiotic Mutants, in Pollen, Biology and Implications for Plant Breeding, eds Mulcahy D. L. Mulcahy Bergamini G. Ottaviano E. (New York, NY: Elsevier), 311–316

[B60] Pérez-Tornero O. Córdoba F. Moreno M. Yuste L. Porras I. (2012). Classic methods and biotechnical tools in lemon breeding, preliminary results. Acta Hortic. 928, 259–263. 10.17660/ActaHortic.2012.928.32

[B61] Ramanna M. Jacobsen E. (2003). Relevance of sexual polyploidization for crop improvement–a review. Euphytica 133, 3–8. 10.1023/A:1025600824483

[B62] Recupero G. R. Russo G. Recupero S. (2005). New promising citrus triploid hybrids selected from crosses between monoembryonic diploid female and tetraploid male parents. HortScience 40, 516–520. Available online at: http://hortsci.ashspublications.org/content/40/3/516.abstract

[B63] Rouiss H. Cuenca J. Navarro L. Ollitrault P. Aleza P. (2017). Tetraploid citrus progenies arising from FDR and SDR unreduced pollen in 4x X 2x hybridizations. Tree Genet. Genomes 13, 10 10.1007/s11295-016-1094-8

[B64] Spiegel-Roy P. Vardi A. Yaniv Y. Fanberstein L. Elhanati A. Carmi N. (2007). ‘Ayelet’ and ‘Galya’, New seedless lemon cultivars. HortScience 42, 1723–1724. Available online at: http://hortsci.ashspublications.org/content/42/7/1723.full

[B65] Starrantino A. Recupero G. (1981). Citrus hybrids obtained *in vitro* from 2x females × 4x males, in Proceedings of 4th International Citrus Congress, Vol 1. (Tokyo: International Society of Citriculture), 31–32.

[B66] Stift M. Berenos C. Kuperus P. Van Tienderen P. (2008). Segregation models for disomic, tetrasomic and intermediate inheritance in tetraploids: a general procedure applied to Rorippa. (Yellow Cress). microsatellite data. Genetics 179, 2113–2123. 10.1534/genetics.107.085027 18689891 PMC2516083

[B67] Tai G. C. C. (1986). Biometrical genetical analysis of tetrasomic inheritance based on matings of diploid parents which produce 2n gametes. Heredity 57, 315–317. 10.1038/hdy.1986.128

[B68] Uzun A. Gulsen O. Kafa G. Seday U. (2008). ‘Alata’, ‘Gulsen’, and ‘Uzun’ Seedless Lemons and ‘Eylul’ Early-maturing Lemon. HortScience 43, 1920–1921. Available online at: http://hortsci.ashspublications.org/content/43/6/1920.full.pdf+html

[B69] Viloria Z. Grosser J. W. (2005). Acid citrus fruit improvement via interploid hybridization using allotetraploid somatic hybrid and autotetraploid breeding parents. J. Am. Soc. Hortic. Sci. 130, 392–402. Available online at: http://journal.ashspublications.org/content/130/3/392.full.pdf+html

[B70] Wakana A. Iwamasa M. Uemoto S. (1982). Seed development in relation to ploidy of zygotic embryo and endosperm in polyembryonic Citrus. In Proc. Int. Soc. Citriculture. 1, 35–39.

[B71] Xie K. Wang X. Biswas M. K. Liang W. Xu Q. Grosser J. W.. (2014). 2n megagametophyte formed via SDR contributes to tetraploidization in polyembryonic ‘Nadorcott’ tangor crossed by citrus allotetraploids. Plant Cell Rep. 33, 1641–1650. 10.1007/s00299-014-1643-2 24972825

[B72] Youds J. L. Mets D. G. McIlwraith M. J. Martin J. S. Ward J. D. Oneil N. J.. (2010). RTEL-1 enforces meiotic crossover interference and homeostasis. Science 327, 1254–1258. 10.1126/science.1183112 20203049 PMC4770885

[B73] Younis A. Hwang Y. Lim K. (2014). Exploitation of induced 2n-gametes for plant breeding. Plant Cell Rep. 33, 215–223. 10.1007/s00299-013-1534-y 24311154

